# Radiomic-Based Quantitative CT Analysis of Pure Ground-Glass Nodules to Predict the Invasiveness of Lung Adenocarcinoma

**DOI:** 10.3389/fonc.2020.00872

**Published:** 2020-08-11

**Authors:** Fangyi Xu, Wenchao Zhu, Yao Shen, Jian Wang, Rui Xu, Chooah Outesh, Lijiang Song, Yi Gan, Cailing Pu, Hongjie Hu

**Affiliations:** ^1^Department of Radiology, Sir Run Run Shaw Hospital, Zhejiang University School of Medicine, Hangzhou, China; ^2^Department of Radiology, Yinzhou Hospital Affiliated With the School of Medicine of Ningbo University, Ningbo, China; ^3^Department of Radiology, Tongde Hospital of Zhejiang Province, Hangzhou, China; ^4^DUT-RU International School of Information Science & Engineering, Dalian University of Technology, Dalian, China; ^5^DUT-RU Co-Research Center of Advanced ICT for Active Life, Dalian, China; ^6^Department of Cardiothoracic Surgery, Sir Run Run Shaw Hospital, Zhejiang University School of Medicine, Hangzhou, China; ^7^Department of Pathology, Sir Run Run Shaw Hospital, Zhejiang University School of Medicine, Hangzhou, China

**Keywords:** radiomics, lung cancer, adenocarcinoma, computed tomography, machine learning

## Abstract

**Objectives:** To investigate the performance of radiomic-based quantitative analysis on CT images in predicting invasiveness of lung adenocarcinoma manifesting as pure ground-glass nodules (pGGNs).

**Methods:** A total of 275 lung adenocarcinoma cases, with 322 pGGNs resected surgically and confirmed pathologically, from January 2015 to October 2017 were enrolled in this retrospective study. All nodules were split into training and test cohorts randomly with a ratio of 4:1 to establish models to predict between pGGN-like adenocarcinoma *in situ* (AIS)/minimally invasive adenocarcinoma (MIA) and invasive adenocarcinoma (IVA). Radiomic feature extraction was performed using Pyradiomics with semi-automatically segmented tumor regions on CT scans that were contoured with an in-house plugin for 3D-Slicer. Random forest (RF) and support vector machine (SVM) were used for feature selection and predictive model building in the training cohort. Three different predictive models containing conventional, radiomic, and combined models were built on the basis of the selected clinical, radiological, and radiomic features. The predictive performance of each model was evaluated through the receiver operating characteristic curve (ROC) and the area under the curve (AUC). The predictive performance of two radiologists (A and B) and our radiomic predictive model were further investigated in the test cohort to see if radiomic predictive model could improve radiologists' performance in prediction between pGGN-like AIS/MIA and IVA.

**Results:** Among 322 nodules, 48 (14.9%) were AIS and 102 (31.7%) were MIA with 172 (53.4%) for IVA. Age, diameter, density, and nine meaningful radiomic features were selected for model building in the training cohort. Three predictive models showed good performance in prediction between pGGN-like AIS/MIA and IVA (AUC > 0.8, *P* < 0.05) in both training and test cohorts. The AUC values in the test cohort were 0.824 (95% CI, 0.723–0.924), 0.833 (95% CI, 0.733–0.934), and 0.848 (95% CI, 0.750–0.946) for conventional, radiomic, and combined models, respectively. The predictive accuracy was 73.44 and 59.38% for radiologist A and radiologist B in the test cohort and was improved dramatically to 79.69 and 75.00% with the aid of our radiomic predictive model.

**Conclusion:** The predictive models built in our study showed good predictive power with good accuracy and sensitivity, which provided a non-invasive, convenient, economic, and repeatable way for the prediction between IVA and AIS/MIA representing as pGGNs. The radiomic predictive model outperformed two radiologists in predicting pGGN-like AIS/MIA and IVA, and could significantly improve the predictive performance of the two radiologists, especially radiologist B with less experience in medical imaging diagnosis. The selected radiomic features in our research did not provide more useful information to improve the combined predictive model's performance.

## Background

A new classification for lung adenocarcinoma was proposed in 2011 by the International Association for the Study of Lung Cancer/American Thoracic Society/European Respiratory Society (IASLC/ATS/ERS) (1), which was also issued as the 4th edition WHO lung cancer classification in 2015 (2). According to the new classification, lung adenocarcinoma can be divided into preinvasive lesion, minimally invasive adenocarcinoma (MIA), and invasive adenocarcinoma (IVA), and preinvasive lesion includes atypical adenomatous hyperplasia (AAH) and adenocarcinoma *in situ* (AIS) (1). The improvement of medical technology and the generalization of lung cancer screening project have led more attention to pure ground-glass nodules (pGGNs) detected on computed tomography (CT) images (3, 4).

Approximately 20% of lung adenocarcinoma including AIS, MIA, and even some early-stage IVA could present as pGGNs on CT images (4), which makes it quite difficult for radiologists and clinicians to make a precise diagnosis with conventional radiological parameters like size, density, etc. Kakinuma et al. reported that growth was observed in approximately 10% of pGGNs ≤5 mm, of which 1% would develop into IVA or MIA in their study (5). In another study, 57.8% of pGGNs showed growth during follow-up and 26.3% of them were adenocarcinoma (6). Eguchi et al. examined 124 cases with pGGNs, and 64 pGGNs (51.6%) showed growth during their 2-year follow-up (7). Several previous research revealed that nearly 50% of pGGNs were invasive lesions (3, 8–10). In clinical practice, pGGNs are usually prescribed to be followed up but data above demonstrated that more detailed diagnosis and more individualized management should be made for pGGNs.

Compared with IVA, AIS, and MIA are considered as indolent lung adenocarcinoma because of the excellent prognosis (11, 12). AIS/MIA could be followed up or treated with sublobar resection while more aggressive surgical interventions should be taken for IVA (13, 14). Several previous studies revealed that the 5-year survival rates of AIS and MIA could be 100% and near 100% with a complete resection while that of IVA in stage Ia is no more than 75% (11, 13, 14). Thus, it might provide some guidance for clinical therapeutic decision-making if pGGN-like IVA could be figured out on preoperative CT images.

There were many studies investigating the difference in radiological features among lung adenocarcinoma subtypes. Wang et al. found that the mean CT attenuation and lesion size differed significantly between MIA and non-invasive lesions and internal air bronchograms were more often seen in adenocarcinoma (15). Several investigations reported that the notched signs, spiculations, bubbly lucencies, and rapid volume expansion were more common in IVA (15–17). However, those radiological features could be subtly different because of the small size of pGGN-like adenocarcinoma. Furthermore, the assessment of those parameters tends to be subjective, which could be influenced by radiologists' experience and diagnostic ability. Percutaneous biopsy is one method used in clinical practice to determine the nature of pulmonary nodules, which could provide relatively accurate pathological information. However, percutaneous biopsy is an invasive operation, and patients may have some operation-related complications (18, 19). Considering the heterogeneity in adenocarcinoma, small pieces of tissue obtained by biopsy cannot represent the characteristics of the whole lesion (20). What is more, in some cases, it is difficult to complete biopsy due to patient's physical condition and bad cooperation as well as the location and size of nodules (20, 21). Thus, the accurate diagnosis of pGGNs remains a key point and a challenge in the field of medical imaging diagnosis.

Radiomics is an emerging subject that could extract a large amount of invisible features from medical images for clinical decision-making (20, 22). Radiomics has had remarkable progress in central nervous system malignancies, thoracic imaging diagnosis, discrimination of hepatic mass, and some other diseases (23–26). Chaddad et al. performed retrospective analysis involving 315 patients diagnosed as non-small cell lung cancer (NSCLC) and significant correlation was observed between radiomic features and survival (27). Also, radiomics' promising performance in the distinction of benign and malignant pulmonary nodules and the discrimination of adenocarcinoma subtypes had been validated in several researches (11, 28, 29). However, in most previous radiomic studies, all types of pulmonary nodules including solid and subsolid nodules were recruited as the study population. Few studies focused on the use of machine learning in early-stage lung adenocarcinoma representing as pGGNs, which are usually very difficult to manage. Since the diagnosis of solid components in pulmonary nodules on CT images is relatively uncomplicated while pGGNs remain a big challenge for medical imaging diagnosis, we aimed to explore the potential value of radiomic-based quantitative analysis to predict the invasiveness of pGGN-like adenocarcinoma to establish a comprehensive predictive model for clinical decision-making.

## Materials and Methods

This retrospective study was approved by the institutional review committee of the Sir Run Run Shaw Hospital (No. 20190520-162) with an abstention of informed consents from all the patients involved according to the guidelines of the Council for International Organizations of Medical Science (CIOMS).

### Patients

We reviewed all the materials of 1,610 patients undergoing surgical resection for primary lung adenocarcinoma with complete clinical data and preoperative CT images from January 2015 to October 2017 in Sir Run Run Shaw Hospital, and reinterpreted the preoperative CT images from the Picture Archiving and Communication Systems (PACS) one by one. Clinical data like age, gender, and smoking status of all cases were collected from digital records. Patients who met any one of the following criteria were excluded: (1) nodules with solid components (*n* = 776), (2) nodule diameter >3 cm (*n* = 292), (3) patients with a history of other malignant diseases (*n* = 87), (4) CT images with bad quality (*n* = 70), and (5) patients who accepted thoracic surgical intervention, radiation, or any chemotherapeutics (*n* = 110).

**Figure 1:**
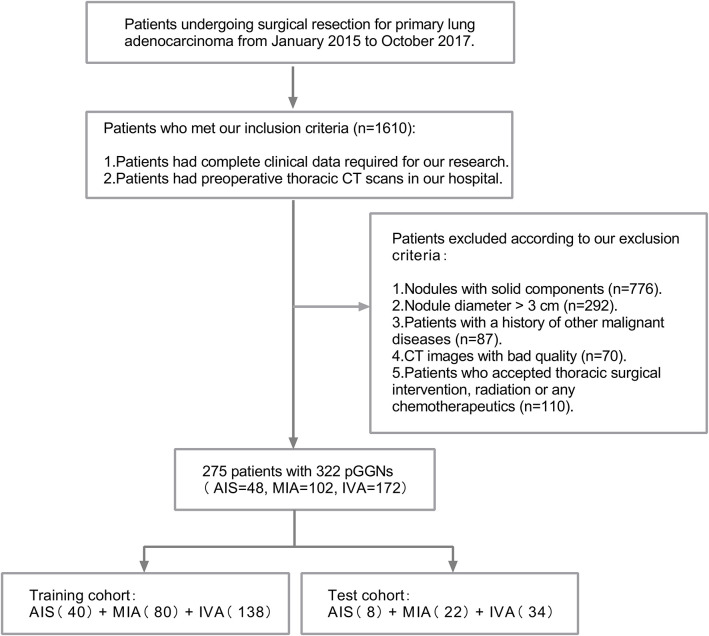
The flowchart of patient selection.

Finally, 275 patients (72 men and 203 women, age range, 25~78 years) with 322 pGGNs (82 men and 240 women, age range, 25~78 years) were enrolled into this retrospective study (detailed in Figure 1 and Table 1). The median time from the last preoperative CT scan to surgery was 6 (0–92) days.

**Table 1:** Analysis of clinical and radiological features of pGGNs in the training and test cohort.

	**Training cohort**				**Test cohort**			
	**Total**	**AIS/MIA**	**IVA**	***P***	**Total**	**AIS/MIA**	**IVA**	***P***
	258	120 (46.5%)	138 (53.5%)		64	30 (46.9%)	34 (53.1%)	
**Gender**								
Male	70 (27.1%)	31 (25.8%)	39 (28.3%)	0.662	12 (18.8%)	3 (10.0%)	9 (26.5%)	0.092
Female	188 (72.9%)	89 (74.2%)	99 (71.7%)		52 (81.3%)	27 (90.0%)	25 (73.5%)	
Age (years)	53 (25, 78)	51 (25, 69)	55 (26, 78)	0.001	54 (30, 72)	51 (31, 72)	55 (30, 72)	0.106^#^
**Smoking history**								
Never	230 (89.1%)	109 (90.8%)	121 (87.7%)	0.417	59 (92.2%)	29 (96.7%)	30 (88.2%)	0.360^*^
Current or ever	28 (10.9%)	11 (9.2%)	17 (12.3%)		5 (7.8%)	1 (3.3%)	4 (11.8%)	
Diameter (cm)	0.94 (0.24, 2.94)	0.76 (0.24, 1.92)	1.13 (0.51, 2.94)	<0.001	0.93 (0.34, 2.04)	0.70 (0.34, 1.41)	1.09 (0.61, 2.04)	<0.001^#^
Density (HU)	−583.0 (−829.2, −122.5)	−615.0 (−801.7, −122.5)	−529.2 (−829.2, −176.3)	<0.001	−525.8 (−763.2, −179.8)	−544.7 (−763.2, −298.9)	−509.0 (−739.1, −179.8)	0.088^#^

# *Two-sample t-test*.

* *Fisher exact probability test*.

*Continuous variables were represented in median (minimum, maximum) without any special statement*.

*pGGNs, pure ground-glass nodules; AIS, adenocarcinoma in situ; MIA, minimally invasive adenocarcinoma; IVA, invasive adenocarcinoma*.

### Histological Evaluation

All surgical specimens were fixed with formalin and stained with hematoxylin–eosin (HE). Two pathologists evaluated all slides using a multi-headed microscope and discussed about the diagnosis until a consensus was reached. According to the classification of lung adenocarcinoma issued in the 4th edition WHO lung cancer classification in 2015, each nodule was classified as AIS, MIA, and IVA (26, 30). Each histological pattern presented in targeted lesion including lepidic, acinar, solid, papillary, and micropapillary patterns was recorded in 5% increments (30, 31).

### Image Acquisition

All the plain CT images were obtained using multidetector computed tomography scanners (Siemens SOMATOM Definition Flash, Siemens FORCE CT, Siemens Sensation 16, Siemens Definition AS 40, and GE LightSpeed VCT). The protocol parameters for each scanning were detailed in Supplemental Table 1. Plain spiral acquisitions were obtained from thoracic inlet to lung bases on patients accepting breath-hold training. Images were reconstructed using a standard reconstruction kernels in lung window settings (mean, −500 HU; width, 1,500 HU). All images underwent multi-planar reconstruction (MPR) including coronal and sagittal reconstruction utilizing the post-processing station.

### Nodule Analysis and Segmentation

All transverse CT images were interpreted jointly by two radiologists who were both blind to the clinical and pathological information of all cases (radiologist A, with 10 years' experience in thoracic imaging diagnosis; radiologist B, with 2 years' experience in medical imaging diagnosis) on our professional reading screen. Conventional quantitative radiological features that were widely used in clinical diagnosis involving diameter (cm) and density (Hounsfield Unit, HU) were determined for each nodule. Nodule diameter was measured on the average of long- and short-axis diameters, both of which should be obtained on the same transverse revealing the greatest dimensions. The nodule density was measured at three different parts of each nodule avoiding vessels and bronchus and the mean value of the three results was calculated. The mean value of diameter and density measured by the two radiologists was calculated for our study.

Segmentation data consisted of all the 322 pGGNs. Plain CT images were loaded into 3D-slicer (http://www.slicer.org) (32), an open source image processing software, implemented with in-house algorithm for automatic nodule detection and segmentation. Radiologist B would verify the regions of interest (ROIs) of automatic segmentation and made some modifications when the ROIs were not satisfactory. Radiologist A would have a second review for the results of radiologist B's semi-automatic segmentation. A consensus would be achieved via negotiation between two radiologists for each case when meeting a collision on reviewing. While modifying ROIs, two radiologists would delineate manually around the nodule boundary on each section avoiding the bronchus and vessels as much as they could.

### Radiomic Feature Extraction and Predictive Models Building

Segmentation data were analyzed with Pyradiomics to extract radiomic features describing tumor phenotypes (33). All the segmentation data had a voxel resampling of 0.7 × 0.7 × 0.7 mm^3^ for standardization to reduce the impact from the heterogeneity of image acquisition. In the end, we obtained nine types totaling 960 radiomic features for each nodule, which have been listed in Supplemental Table 2. Features are commonly grouped as follows: (1) first-order statistical features: these describe the voxel intensity distribution in the delineated ROI. They are usually calculated on the basis of the intensity histogram, including energy, entropy, standard deviation, skewness, kurtosis, uniformity, mean, minimum, and maximum intensity values and so on (20, 26). (2) Shape-based features: descriptors of the two- and three-dimensional size and shape of the ROI. (3) Textural features: these contain gray level co-occurrence matrix (GLCM), gray level run length matrix (GLRLM), gray level size zone matrix (GLSZM), neighboring gray size zone matrix (NGZDM), and gray-level dependence matrix (GLDM). They are computed on the analysis of the three-dimensional directions within the tumor and the consideration of the spatial location of each voxel in the ROI (26). (4) Transformed features: features in the first and third groups extracted from images applied with a series of wavelet or Laplacian-of-Gaussian filtration.

Random forest (RF) and support vector machine (SVM) with cross-validation (CV) was used for radiomic feature selection and predictive model building to distinct pGGN-like IVA from indolent adenocarcinoma (AIS/MIA). Multivariate models were made in training cohort and were tested in a separate test cohort. All pGGNs were split into training and test cohorts randomly by a ratio of 4:1. Three predictive models were created in our research: (1) Conventional (selected clinical and radiological quantitative features), (2) Radiomic (selected radiomic features), and (3) Combined (selected conventional and radiomic features) predictive models. Subsequently, a binary analysis in which pGGN-like IVA was set as positive while AIS/MIA was thought as negative was applied to compare the predictive performance between radiomic predictive model and two radiologists (A and B) in the test cohort. Two weeks later, two radiologists, knowing the performance of our radiomic predictive model and its diagnosis for each pGGN in the test cohort, reevaluated all pGGNs in the test cohort.

### Statistical Analysis

All the statistical analysis was applied using SPSS 25.0 (IBM, Armonk, NY, USA) and MedCalc 15.8 (MedCalc Software, Acacialaan 22, Ostend, Belgium). Tables and figures in our study were made with GraphPad Prism 5 (GraphPad Software Inc., San Diego, CA, USA) and Microsoft Office 2019 (Microsoft, Redmond, WDC, USA).

Thirty pGGNs were selected randomly to test the repeatability of nodule diameter and density measurement. Radiologist A and B did the measurement work of those 30 pGGNs, respectively. Two weeks later, radiologist B measured the diameter and density of these 30 pGGNs, again according to the same measurement criteria. Inter-/intra-observer correlation coefficient (ICC) was calculated for repeatability assessment.

For the assessment of clinical, quantitative radiological, and selected radiomic features, chi-square test or Fisher exact probability test was utilized for categorical variables. Two-sample *t*-test was adopted, if the continuous variables met the normal test and variance homogeneity test; otherwise, Wilcoxon signed-rank test was used. Predictive power of each predictive model was evaluated using receiver operating characteristic (ROC) curve and area under the curve (AUC). Models with an AUC > 0.50 and a *P* < 0.05 were thought to be predictive. McNemar's test and Kappa analysis were used to compare the binary diagnosis of two radiologists and radiomic predictive model.

## Results

The measurement of nodule diameter and density between senior radiologist A and junior radiologist B was highly consistent (ICC > 0.9, *P* < 0.05). Two weeks later, radiologist B did the measurement for the 30 selected pGGNs, and the ICC values were up to 0.955 (*P* < 0.05) and 0.984 (*P* < 0.05) for diameter and density measurement.

### Clinical Data and Conventional Image Features

A total of 322 pGGNs were recruited into this study with 80% in the training cohort and 20% in the test cohort. The analysis of clinical and quantitative radiological features in the training and test cohort were listed in Table 1. In the training cohort, the median age was 53 years (age range, 25–78 years) and the majority of cases were female (72.9%) with 28 (10.9%) having a smoking history. In the test cohort, 52 (81.3%) were female with a median age of 54 years (age range, 30–72 years) and 59 (92.2%) never smoke. Diameter showed statistical discrepancy between AIS/MIA and IVA in both training and test cohort (*P* < 0.001) while nothing significantly different existed in gender and smoking status between the two groups. Age and density exhibited evident difference in training cohort between AIS/MIA and IVA (*P* = 0.01 and *P* < 0.001), but no significant difference (*P* > 0.05) appeared between the two groups in test cohort.

### Radiomic Feature Selection and Predictive Model Building

**Figure 2:**
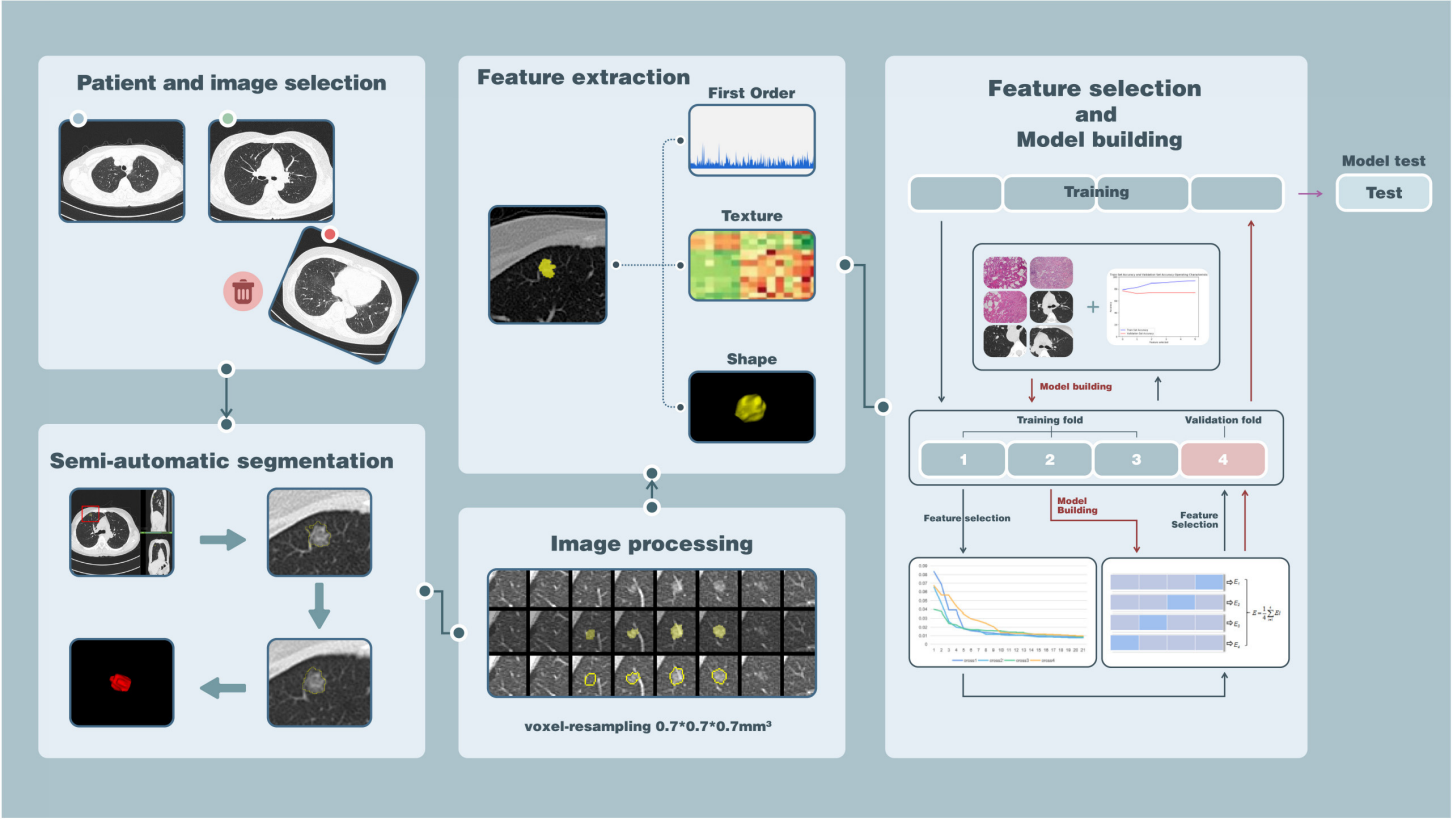
The flowchart of the whole study.

A RF algorithm with 4-fold cross-validation was taken to calculate the contribution value of each radiomic feature in the training cohort for the prediction of pGGN-like IVA from AIS/MIA. Predictive model building was performed using SVM also combined with 4-fold cross-validation. All the extracted radiomic features were listed in descending order by the contribution value for the classifier and were added one by one as the input for the SVM model training in each iteration of cross-validation calculation (detailed in Figure 2). In the process of gradual accumulation of model training, the overall accuracy of the training cohort was recorded. When sequencing extracted radiomic features by their contribution value for the classifier in each iteration, we found that the first 20 radiomic features contributed much more than other features whose contribution values were <0.01 and even close to 0. Meanwhile, the first 20 radiomic features could increase the classifying performance dramatically while inclusion of extra features did not make a big difference to the performance of the SVM classifier in each iteration. To improve the generalization of our predictive model, only radiomic features appearing more than three times in the first 20 of contribution value rank in four iterations of the 4-fold cross validation were selected for final model building. Those radiomic features that were used for final predictive model building incorporated four from GLCM, four from GLSZM, and one from GLRLM. All the nine features are detailed in Table 2.

**Table 2:** Nine selected radiomic features.

	**Selected radiomic feature**	**Radiomic group**	**Filter associated**
R1	Maximum probability	GLCM	wavelet-LLL
R2	Joint entropy	GLCM	None
R3	Sum entropy	GLCM	wavelet-LLL
R4	Joint energy	GLCM	None
R5	Gray level Non-uniformity	GLSZM	log-sigma-1-0-mm-3D
R6	Gray level Non-uniformity	GLSZM	wavelet-LLH
R7	Gray level Non-uniformity	GLSZM	log-sigma-3-0-mm-3D
R8	Size zone Non-uniformity	GLSZM	None
R9	Low gray level run emphasis	GLRLM	log-sigma-3-0-mm-3D

*GLCM, gray level co-occurrence matrix; GLSZM, gray level size zone matrix; GLRLM, gray level run length matrix*.

**Figure 3:**
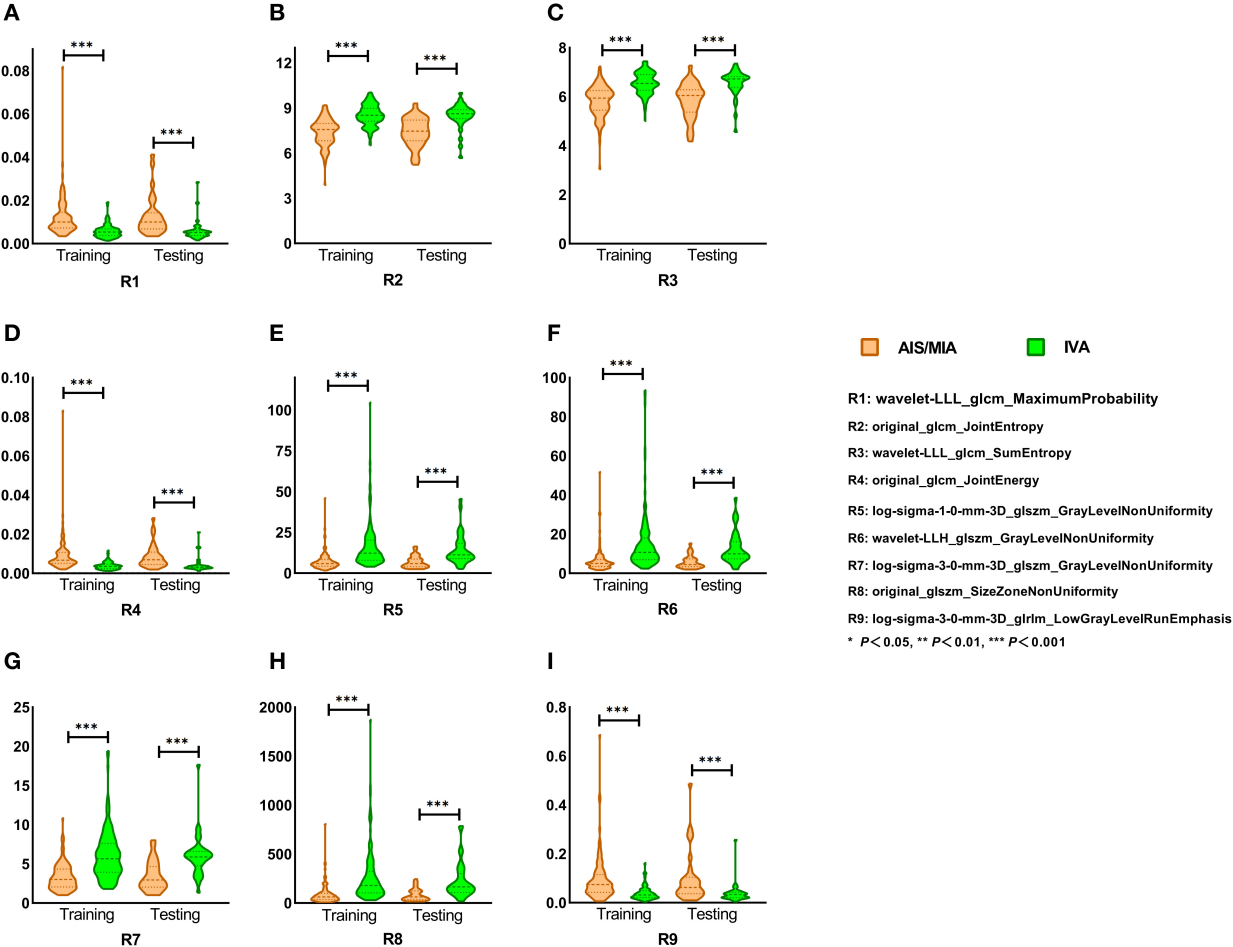
**(A–I)** Selected radiomic features in training and test cohort. Nine selected radiomic features showed significant difference between AIS/MIA and IVA in both training and test cohorts. Maximum Probability, Joint Energy, and Low Gray Level Run Emphasis had larger median values for AIS/MIA, while the median values of Joint Entropy, Sum Entropy, Gray Level Non-Uniformity, and Size Zone Non-uniformity were higher for IVA in both training and test cohorts.

We first investigated whether the nine advanced radiomic features could discriminate IVA from indolent adenocarcinoma (AIS/MIA) representing as pGGNs. Figure 3 shows the comparison of those nine radiomic features for the distinction of pGGN-like AIS/MIA and IVA in training and test cohorts. All the selected radiomic features revealed significant difference between AIS/MIA and IVA in both training and test cohorts (*P* < 0.001). Among the nine radiomic features, three (Maximum Probability, Joint Energy, and Low Gray Level Run Emphasis) had larger median values for AIS/MIA while the median values of six other features (Joint Entropy, Sum Entropy, Gray Level Non-Uniformity, and Three Different Filtered Size Zone Non-Uniformity) were higher for IVA than that for AIS/MIA in both training and test cohorts (detailed in Supplemental Table 3).

**Figure 4:**
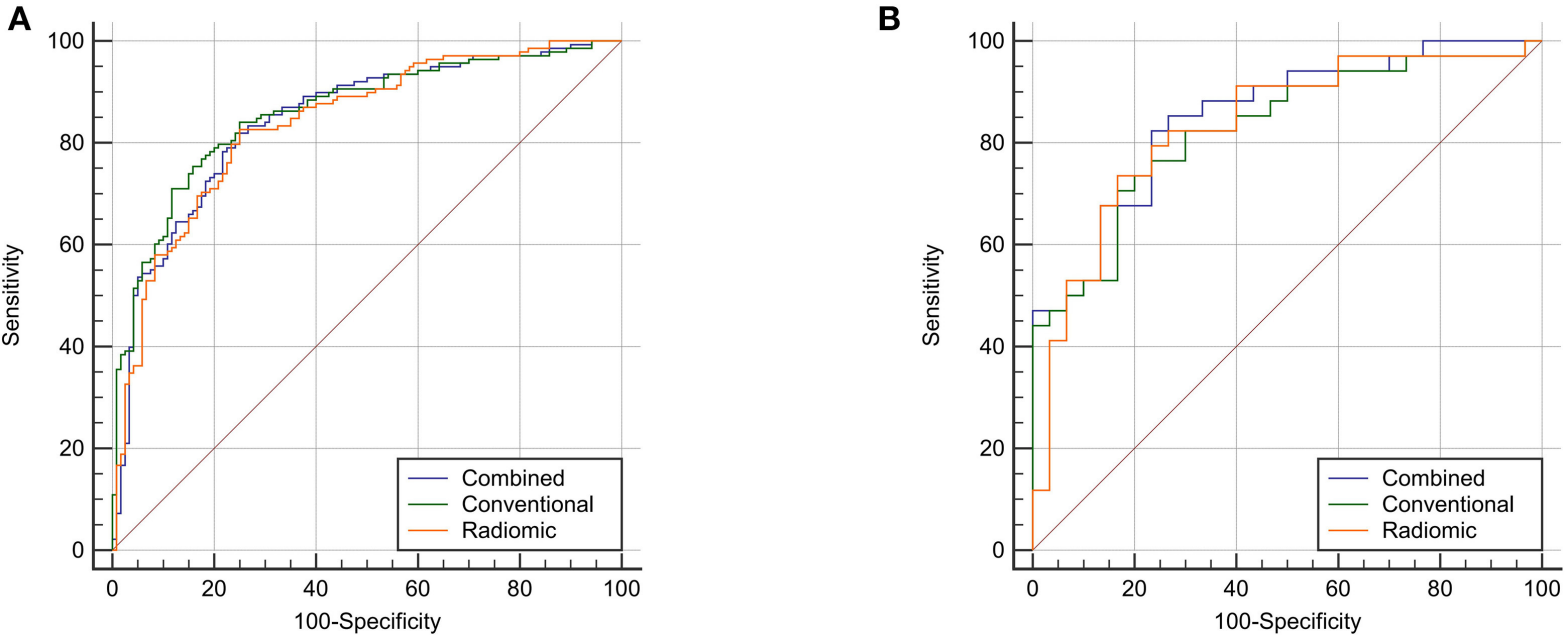
The ROC analysis of the three different predictive model. Three predictive models presented good performance in discrimination between pGGN-like IVA and AIS/MIA. **(A)**, the predictive performance of three models in the 693 training cohort; **(B)**, the predictive performance of three models in the testing cohort.

**Figure 5:**
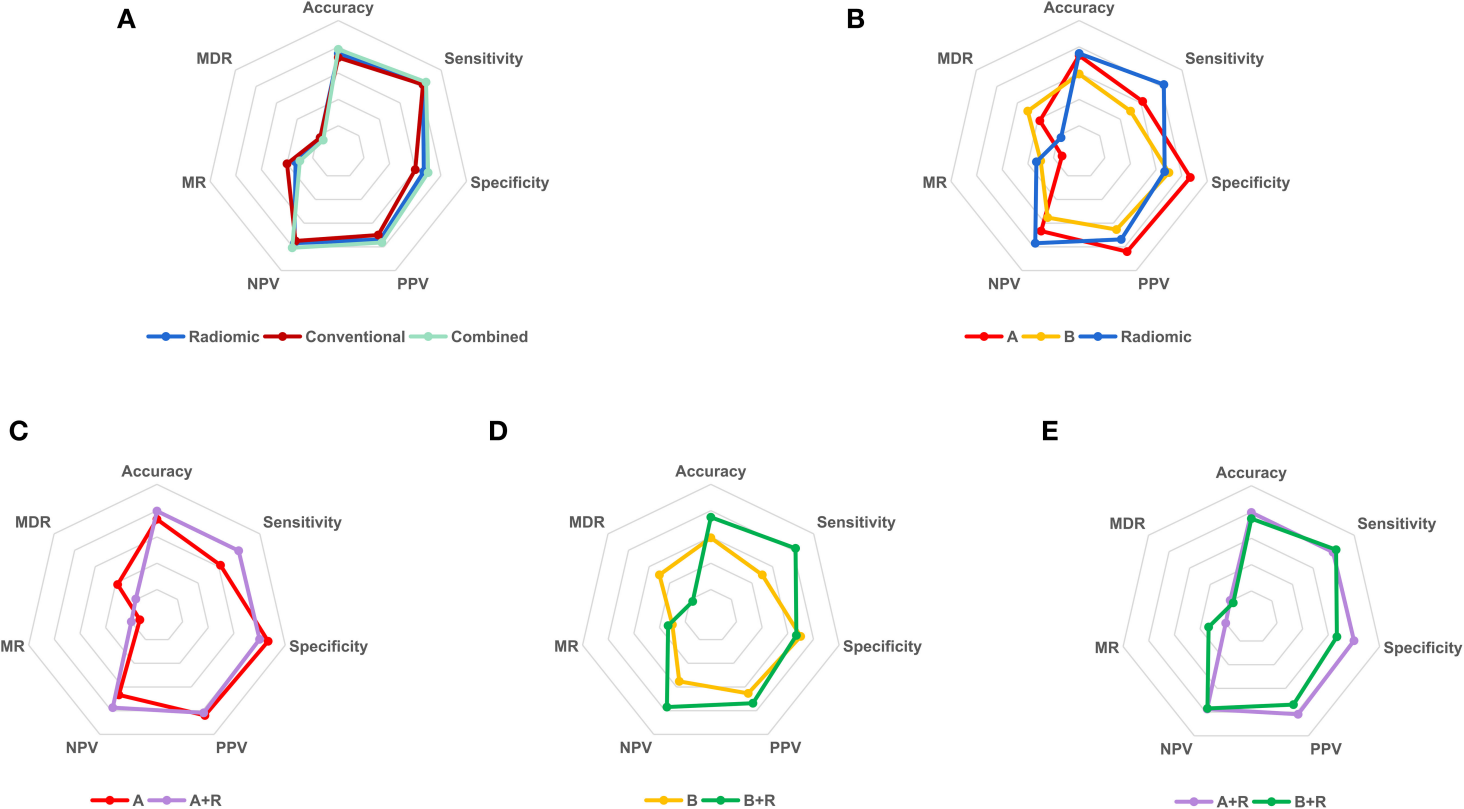
The binary diagnosis of predictive models and two radiologists. (A), the first binary diagnosis of the senior radiologist A with 10-year experience in thoracic imaging diagnosis in test cohort; (B), the first binary diagnosis of the junior radiologist B with 2-year experience in medical imaging diagnosis in test cohort; R, the binary diagnosis of radiomic predictive model in test cohort; A+R, the second binary diagnosis of radiologist A in test cohort with the aid of radiomic predictive model; B+R, the second binary diagnosis of radiologist B in test cohort with the aid of radiomic predictive model. PPV, positive predictive value; NPV, negative predictive value; MR, misdiagnosis rate; MDR, missed diagnosis rate.

Multivariate predictive models were created for each set of features, involving conventional (age, diameter, and density), radiomic (nine predictive features), and the combined (conventional and selected radiomic features) sets. The three different models presented good predictive power (AUC > 0.8, *P* < 0.05) in both training and test cohorts as shown in Figure 4, Supplemental Table 4. Then, DeLong's test (34, 35) was applied to complete the pairwise predictive performance comparison among the three models, in which no significant difference was observed (*P* > 0.05). Figure 5 showed comprehensive parameters including accuracy, sensitivity, specificity, positive predictive value (PPV), negative predictive value (NPV), misdiagnosis rate (MR), and missed diagnosis rate (MDR) of the three predictive models' and two radiologists' binary diagnosis in the test cohort (detailed in Supplemental Table 5). The accuracy was 76.56, 71.88, and 78.13% for radiomic, conventional, and combined predictive models but no big difference was noted in comprehensive assessment among the three models (Figure 5), which was consistent with the results of DeLong's test above. In a word, no matter what features were used for model training (conventional or radiomic features), predictive models built with machine learning algorithm could predict pGGN-like IVA from AIS/MIA well. The combination of conventional and radiomic features could further improve the diagnosis accuracy of predictive model, but the improvement was not statistically significant in our study.

To investigate whether the radiomic predictive model could help radiologists improve their predictive performance, we then compared the diagnosis of radiomic predictive model and two radiologists (Figure 5, Table 3). Radiologist A performed better than radiologist B with higher diagnostic accuracy, sensitivity, specificity, PPV, and NPV. Either accuracy or sensitivity, radiomic predictive model outperformed radiologist A with the cost of decreased specificity. Significant difference was observed between the binary diagnosis of radiomic predictive model and that of two radiologists (A vs. R, χ^2^ = 7.563, *P* = 0.004, B vs. R, χ^2^ = 4, *P* = 0.043). Generally speaking, Radiomic predictive model showed better performance than two radiologists in the prediction between pGGN-like IVA and AIS/MIA. Two radiologists dramatically improved their diagnostic accuracy to 79.69 and 75.00% with the aid of radiomic predictive model (A vs. A+R, χ^2^ = 4.9, *P* = 0.021, B vs. B+R, χ^2^ = 5.042, *P* = 0.023). The comparison of the second diagnosis of two radiologists revealed that when having the guidance from radiomic predictive model, no statistical difference existed between two radiologists in prediction of pGGN-like IVA and AIS/MIA (A+R vs. B+R, χ^2^ = 1.455, *P* = 0.227).

**Table 3:** Performance comparison between radiomic predictive model and two radiologists in test cohort.

	**McNemar's test**		**Kappa analysis**	
	**χ^**2**^**	***P***	**κ**	***P***
A vs. B	0	1.000	0.316	0.018
A vs. R	7.563	0.004	0.517	<0.001
B vs. R	4	0.043	0.241	0.070
A vs. A+R	4.9	0.021	0.690	<0.001
B vs. B+R	5.042	0.023	0.275	0.022
A+R vs. B+R	1.455	0.227	0.654	<0.001

*A, the first binary diagnosis of the senior radiologist A with 10-year experience in thoracic imaging diagnosis in test cohort; B, the first binary diagnosis of the junior radiologist B with 2-year experience in medical imaging diagnosis in test cohort; R, the binary diagnosis of radiomic predictive model in test cohort; A+R, the second binary diagnosis of radiologist A in the test cohort with the aid of radiomic predictive model; B+R, the second binary diagnosis of radiologist B in the test cohort with the aid of radiomic predictive model*.

## Discussion

When it comes to pure ground-glass pulmonary nodules, clinicians tend to choose follow-up as their first choice for management. However, according to previous studies (4, 5, 31), a certain proportion of IVA that needs surgical treatment could be pGGNs on CT scans. If a pGGN-like IVA was misdiagnosed as a benign lesion or indolent adenocarcinoma and was given a prescription of follow-up, it might progress during the interval or even metastasize and miss the optimal time for surgical intervention. Conventional radiological features like lobulated signs, spiculations, bubble lucencies, and pleura traction have been demonstrated to be helpful to differentiate the malignancy of pulmonary nodules and the invasiveness of lung adenocarcinoma (36, 37). However, pGGN-like lung adenocarcinoma tends to be in small volume with a large similarity in morphological characteristics and the assessment of the conventional radiological features is easy to be affected by the subjectivity of doctors, it remains a challenge to make a precise judgement for pGGNs without surgical intervention. Percutaneous biopsies may be helpful in determining the nature of pulmonary nodules. Nevertheless, it is an invasive tissue extraction method with the possibility of postoperative complications, and due to the tumor heterogeneity, it is not persuasive to represent the characteristics of the entire lesion with only a tiny tissue (20, 38). In some cases, because of nodules' location and size, percutaneous biopsies may not be a good choice for diagnosis. Thus, the diagnosis of pGGNs remains a thorny point for clinical research.

Heidinger et al. reported that two-dimensional diameter could provide enough information for pulmonary nodule risk classification in their quantitative analysis based on CT images (4, 39). In our study, the diameter of pGGNs showed a significantly different distribution between IVA and indolent adenocarcinoma (AIS/MIA), which was consistent with previous reports. Kitami et al. acclaimed that almost all the pGGNs with a diameter <10 mm and a density of no more than −600 HU were demonstrated to be preinvasive lesions in their study (40). The medians of density in our training and test cohort were −583.0 and −525.8 HU, both slightly higher than −600 HU, which might have something to do with the difference in lung adenocarcinoma classification. We classified MIA into indolent adenocarcinoma, which might lead to the increase in density medians.

Radiomics is a new quantitative image analysis approach that allows thorough exploration in medical images and has attracted more and more attention in the field of medicine in recent years. In this study, we obtained plenty of quantitative radiomic features (960 for each nodule) from routine thoracic CT images using machine learning techniques and completed the quantitative analysis of pGGN-like adenocarcinoma classification on the basis of conventional clinical, quantitative radiological, and selected radiomic features. Nine selected radiomic features demonstrated good performance in distinction of the IVA and indolent adenocarcinoma representing pGGNs on unenhanced CT images. Nine selected radiomic features in our study consisted of four GLCM-based features, four GLSZM-based features, and one GLRLM-based feature, which were analyzed as follows:

(1) Four radiomic features from GLCM: Maximum Probability, Joint Entropy, Sum Entropy, and Joint Energy. GLCM was used to compare the gray level correlation between two points in a certain distance in a spatial position, which reflects the comprehensive information about pixel distribution including direction, distance, gray value, and the pattern of gray level arrangement (41, 42). Maximum Probability refers to a pair of pixels with the highest frequency in a GLCM (43, 44). Entropy is a parameter describing the complexity of an image, which means the larger the entropy value of an image is, the more complex the image is (43). Energy is related to the uniformity of gray level distribution and the roughness of image texture with a larger energy indicating a more regular and more stable texture (42, 43). In our study, Maximum Probability and Joint Energy got higher median values for AIS/MIA in the training as well as test cohort while Joint Entropy and Sum Entropy in IVA were higher than that in AIS/MIA in both training and test cohort. This might have something to do with that AIS/MIA tend to be homogeneous, which results in a higher probability of finding pixels with same distribution pattern in AIS/MIA and the different entropy and energy values between AIS/MIA and IVA.

(2) Four radiomic features from GLSZM and one from GLRLM: Gray Level Non-Uniformity filtered with LOG or wavelet algorithm, Size Zone Non-Uniformity, and Low Gray Level Run Emphasis. GLSZM refers to the number of pixels that share the same gray level intensity and the same arrangement pattern in an image while GLRLM calculates the number of pixels with the same gray level value and distribution pattern in a certain direction (45–47). Gray Level Non-Uniformity and Size Zone Non-Uniformity indicate the variability of gray level and size zone volumes in an image, with a higher value referring to more heterogeneity in ROIs (46, 47). In our study, the median values of Gray Level Non-Uniformity and Size Zone Non-Uniformity were higher for IVA, which might be related to the fact that IVA tends to be more heterogeneous. Low Gray Level Run Emphasis analyzes the distribution of low gray level values in an image (47). The homogeneity and relatively lower average density of AIS/MIA might lead to the higher median value of Low Gray Level Run Emphasis for AIS/MIA in our study.

Chen et al. picked 76 features meaningful for the distinction of malignancy of pulmonary nodules from 750 extracted radiomic features and built a predictive model whose accuracy was up to 84% using four selected advanced features (20). Yagi et al. carried out the texture analysis of high-resolution computed tomography (HRCT) and found that 90th percentile and entropy performed well in discrimination between AIS/MIA and IVA with an AUC value of 0.90 (95% CI: 0.84–0.95) (13). Three different predictive models set with clinical, radiological, and nine selected radiomic features from 960 features extracted from unenhanced CT images in our study all presented good predictive power in the discrimination between AIS/MIA (indolent adenocarcinoma) and IVA (AUC > 0.8, *P* < 0.05). She et al. extracted radiomic features from radiological data of 402 cases (207 for training and 195 for test) diagnosed with lung adenocarcinoma and selected five meaningful radiomic features to build a predictive model that outperformed significantly the predictive model only built with conventional radiological features including nodule diameter for the discrimination between IVA and AIS/MIA (11). However, no apparent difference existed between conventional and radiomic predictive models in our study (*P* > 0.05). Combining conventional and radiomic features could improve the AUC value of combined predictive model, but it was not statistically significant. This might be caused by the difference of our study population. All types of pulmonary nodules including solid and subsolid nodules, which have heterogeneous internal density in lesions, were used for She's study. Therefore, compared with conventional radiological features such as diameter and density, radiomic features could more thoroughly analyze the variability and distribution of gray level intensity in ROI, which would provide more valuable information for improvement in diagnostic performance of predictive models. However, the relatively obscure variability of gray level intensity in pGGNs might result in the limited reference value for radiomic features in prediction between pGGN-like IVA and AIS/MIA, which potentially led to the similar diagnostic performance among our three predictive models. Nevertheless, predictive models built using either radiomic or conventional radiological features presented good performance in distinction between pGGN-like IVA and AIS/MIA, which further confirmed the possibility of machine learning methods for the differentiation of the invasion of pGGN-like lung adenocarcinoma. In conclusion, the predictive models established in our study could still provide certain guidance for clinicians to make accurate diagnosis.

**Figure 6:**
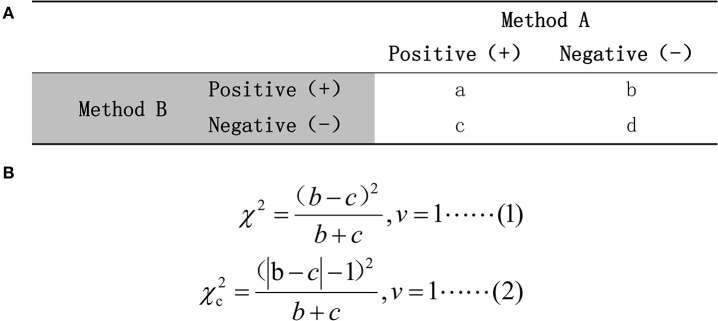
**(A)** The mechanism of McNemar's test. **(B)** The calculation formula for χ^2^-value. Formula (1) would be used if (*b* + *c*) ≥40; otherwise, formula (2) should be chosen.

To assess whether the radiomic predictive model could improve radiologists' performance in diagnosis of pGGN-like lung adenocarcinoma, we further compared the dichotomous diagnosis of the radiomic predictive model and two radiologists. There was a dramatic difference between radiologist A and radiologist B in the values of diagnostic accuracy, sensitivity, and specificity. The Kappa analysis revealed bad consistency between the results of the two radiologists (κ = 0.316, *P* = 0.018) while McNemar's test between that of two radiologists showed no significant difference (*P* < 0.05). This was related to the mechanisms of two statistical methods. McNemar's test only compares results with collision in two diagnostic methods instead of using comprehensive data acquired in a study while Kappa analysis calculates the consistency in all data (48–50). Figure 6 shows the mechanism of McNemar's test and the formula for calculating the value of χ^2^. A small (*b* – *c*) leads to a small χ^2^-value, which results in a *P-*value of more than 0.05 no matter whether the data have actual clinical significance or not. In our test cohort, 32.8% (21/64) of the pGGNs had diverse diagnoses from two radiologists, while the value of (*b* – *c*) is only 1, which resulted in a value of 0 for χ^2^. In this special situation, McNemar's test and Kappa analysis should be combined with actual data distribution to complete the comparison of two radiologists' diagnosis. The comprehensive analysis showed that the diagnostic ability of senior radiologist A was higher than that of junior radiologist B and the radiomic predictive model outperformed two radiologists. When having the diagnosis of the radiomic predictive model for reference, two radiologists could significantly improve their performance in prediction pGGN-like IVA from AIS/MIA. What is more, no significant difference existed between the second diagnosis from two radiologists with the aid of the radiomic predictive model. The predictive model built using selected radiomic features in our research could obviously improve the ability of radiologists in prediction between pGGN-like IVA and AIS/MIA, especially for the junior radiologist; it could help radiologist B reach the level of the senior radiologist A's diagnostic ability, which had certain potential clinical meaning.

There were also some limitations in our study. First, this was a retrospective study in which all the cases were sorted according to rigorous exclusion criteria. There was certain inherent selection bias in it. Meanwhile, 322 pGGNs were not large enough for this quantitative study, compared with 960 features for each nodule. Further data collection including data from other clinic centers would be done to evaluate these models' predictive reproducibility. Second, the feature selection driven by restrictive algorithm (<1% features remaining after) might lead to a certain loss of potential predictive features for distinction. Despite the significant feature reduction, we were still able to find predictive features with high robustness. Third, limitations of this trial included the lack of standardization in image acquisition completed on various CT scanners. A voxel-resampling was applied to reduce the influence from the variability in image acquisition protocols in our study, but the above problem may still have a certain impact on the feature selection and model building. Thus, the standardization of image acquisition and establishment of database with high quality are urgently required for radiomic research. Finally, we chose a semi-automatic method to complete the pGGN segmentation. Though consistent segmentation for each pGGN had been reached through negotiation by two radiologists, there was still some interobserver difference existing in this procedure. A reliable automatic segmentation algorithm that can be applied in clinical practice is still to be developed.

## Conclusion

Nine selected radiomic features in our research showed different distribution between IVA and AIS/MIA, which could provide some guidance for clinical practice. The predictive models established using conventional clinical, radiological, or radiomic features could help to distinguish the invasiveness of pGGN-like lung adenocarcinoma, but radiomic features could not offer more meaningful information to improve the performance of the combined model created in our study. The diagnostic performance of the radiomic predictive model established in our study was better than that of the two radiologists, and the predictive model could provide auxiliary information for radiologists (especially for junior radiologists) to improve their diagnostic ability in discrimination between pGGN-like IVA and AIS/MIA.

## Data Availability Statement

The datasets generated for this study are available on request to the corresponding author.

## Ethics Statement

This study was approved by the Institutional Review Committee of the Sir Run Run Shaw Hospital.

## Author Contributions

FX and WZ conceived and designed this study. FX, YS, and CP applied the data collection for this study. FX and YS did the data reproof work during the manscript review and now they are collecting new data for further exploration. FX and JW did the segmentation work for each pGGN recruited for this study. WZ and RX contributed to the algorithm design for this study. YG completed the pathological interpretation of all pGGNs in this study. FX drafted the manuscript. LS and CQ and HH contributed equally to the manuscript review.

## Conflict of Interest

The authors declare that the research was conducted in the absence of any commercial or financial relationships that could be construed as a potential conflict of interest.
