# Corrigendum: Radiomic-Based Quantitative CT Analysis of Pure Ground-Glass Nodules to Predict the Invasiveness of Lung Adenocarcinoma

**DOI:** 10.3389/fonc.2020.608365

**Published:** 2020-10-30

**Authors:** Fangyi Xu, Wenchao Zhu, Yao Shen, Jian Wang, Rui Xu, Chooah Outesh, Lijiang Song, Yi Gan, Cailing Pu, Hongjie Hu

**Affiliations:** ^1^Department of Radiology, Sir Run Run Shaw Hospital, Zhejiang University School of Medicine, Hangzhou, China; ^2^Department of Radiology, Yinzhou Hospital Affiliated With the School of Medicine of Ningbo University, Ningbo, China; ^3^Department of Radiology, Tongde Hospital of Zhejiang Province, Hangzhou, China; ^4^DUT-RU International School of Information Science & Engineering, Dalian University of Technology, Dalian, China; ^5^DUT-RU Co-Research Center of Advanced ICT for Active Life, Dalian, China; ^6^Department of Cardiothoracic Surgery, Sir Run Run Shaw Hospital, Zhejiang University School of Medicine, Hangzhou, China; ^7^Department of Pathology, Sir Run Run Shaw Hospital, Zhejiang University School of Medicine, Hangzhou, China

**Keywords:** radiomics, lung cancer, adenocarcinoma, computed tomography, machine learning

An author name was incorrectly spelled as Chooah Qutesh. The correct spelling is Chooah Outesh.

The authors apologize for this error and state that this does not change the scientific conclusions of the article in any way. The original article has been updated.

