# Supplementary material for: Radiomic-Based Quantitative CT Analysis of Pure Ground-Glass Nodules to Predict the Invasiveness of Lung Adenocarcinoma
Source: Front Oncol. 2020 Aug 11;10:872. doi: 10.3389/fonc.2020.00872 (PMC7432133; doi:10.3389/fonc.2020.00872)
Supplement: Supplementary file 1 [file Data_Sheet_1.PDF]

Supplemental table 1 Protocols for image acquisition.

| | Reconstruction thickness (mm) | Tube voltage (KV) | Tube current (mAs) | Rotation time (s) | Acquisition matrix |
|---|---|---|---|---|---|
| **Siemens SOMATOM Definition Flash** | 2.0 | 120 | smart | 0.5 | 512*512 |
| **Siemens FORCE CT** | 2.0 | 100 | smart | 0.5 | 512*512 |
| **Siemens Sensation16** | 2.0 | 120 | smart | 0.5 | 512*512 |
| **Siemens Definition AS 40** | 2.0 | 120 | smart | 0.5 | 512*512 |
| **Siemens Definition AS 20** | 1.5 | 120 | smart | 0.5 | 512*512 |
| **GE LightSpeed VCT** | 1.25 | 120 | smart | 0.5 | 512*512 |

Supplemental table 2 List of all extracted radiomic features.

| **Feature types** | **n** |
|---|---|
| First order features | 19 |
| 2D/3D Shape features | 26 |
| GLCM based features | 24 |
| GLRLM based features | 16 |
| GLSZM based features | 16 |
| NGZDM based features | 5 |
| GLDM based features | 14 |
| LOG based features | 172 |
| Wavelet based features | 668 |

GLCM, gray level co-occurrence matrix; GLRLM, gray level run length matrix; GLSZM, gray level size zone matrix; NGSZM, neighbouring gray size zone matrix; GLDM, gray level dependence matrix; LOG, Laplacian-of-Gaussian.

Supplemental table 3 Selected radiomic features of pGGNs in training and test cohort.

| | **Training cohort** | | | | **Test cohort** | | | |
|---|---|---|---|---|---|---|---|---|
| | **Total** | **AIS/MIA** | **IVA** | ***P*** | **Total** | **AIS/MIA** | **IVA** | ***P*** |
| **R1** | $7*10^{-3}$ ($1*10^{-3}$, $82*10^{-3}$) | $10*10^{-3}$ ($3*10^{-3}$, $82*10^{-3}$) | $5*10^{-3}$ ($1*10^{-3}$, $19*10^{-3}$) | <0.001 | $6*10^{-3}$ ($2*10^{-3}$, $41*10^{-3}$) | $10*10^{-3}$ ($3*10^{-3}$, $41*10^{-3}$) | $5*10^{-3}$ ($2*10^{-3}$, $29*10^{-3}$) | <0.001 |
| **R2** | 8.1 (3.9, 10.0) | 7.6 (3.9, 9.2) | 8.5 (6.5, 10.0) | <0.001 | 8.3 (5.2, 10.0) | 7.5 (5.2, 9.3) | 8.6 (5.7, 10.0) | <0.001 |
| **R3** | 6.3 (3.0, 7.5) | 5.9 (3.0, 7.2) | 6.5 (5.0, 7.5) | <0.001 | 6.4 (4.2, 7.4) | 6.1 (4.2, 7.3) | 6.7 (4.6, 7.4) | <0.001 |
| **R4** | $5*10^{-3}$ ($1*10^{-3}$, $83*10^{-3}$) | $7*10^{-3}$ ($2*10^{-3}$, $83*10^{-3}$) | $4*10^{-3}$ ($1*10^{-3}$, $12*10^{-3}$) | <0.001 | $4*10^{-3}$ ($1*10^{-3}$, $28*10^{-3}$) | $7*10^{-3}$ ($2*10^{-3}$, $28*10^{-3}$) | $3*10^{-3}$ ($1*10^{-3}$, $21*10^{-3}$) | <0.001 |
| **R5** | 8.2 (1.6, 104.8) | 5.9 (1.6, 46.1) | 12.3 (3.9, 104.8) | <0.001 | 9.0 (2.5, 45.6) | 5.8 (2.5, 16.4) | 11.3 (2.5, 45.6) | <0.001 |
| **R6** | 7.4 (1.6, 93.7) | 5.1 (1.6, 52.0) | 10.7 (2.4, 93.7) | <0.001 | 7.4 (1.9, 38.7) | 4.4 (1.9, 15.4) | 9.9 (2.0, 38.7) | <0.001 |
| **R7** | 4.2 (1.0, 19.4) | 3.0 (1.0, 10.9) | 5.6 (1.8, 19.4) | <0.001 | 5.0 (1.0, 17.) | 3.0 (1.0, 8.0) | 5.9 (1.3, 17.7) | <0.001 |
| **R8** | 108.0 (5.8, 1874.6) | 62.0 (5.8, 809.2) | 179.2 (29.2, 1874.6) | <0.001 | 121.9 (16.6, 786.9) | 62.8 (16.6, 245.5) | 166.3 (17.8, 786.9) | <0.001 |
| **R9** | $43*10^{-3}$ ($4*10^{-3}$, 687*10-3) | $74*10^{-3}$ ($6*10^{-3}$, 687*10-3) | $32*10^{-3}$ ($4*10^{-3}$, 164*10-3) | <0.001 | $39*10^{-3}$ ($4*10^{-3}$, 488*10-3) | $62*10^{-3}$ ($10*10^{-3}$, 488*10-3) | $34*10^{-3}$ ($4*10^{-3}$, 258*10-3) | <0.001 |

Continuous variables were represented in median (minimum, maximum) without any special statement. pGGNs = pure ground glass nodules; AIS = adenocarcinoma in situ; MIA = minimally invasive adenocarcinoma; IVA = invasive adenocarcinoma; R1 = wavelet-LLL_glcm_MaximumProbability; R2 = original_glcm_JointEntropy; R3 = wavelet-LLL_glcm_SumEntropy; R4 = original_glcm_JointEnergy; R5 = log-sigma-1-0-mm-3D_glszm_GrayLevelNonUniformity; R6 = wavelet-LLH_glszm_GrayLevelNonUniformity; R7 = log-sigma-3-0-mm-3D_glszm_GrayLevelNonUniformity; R8 = original_glszm_SizeZoneNonUniformity; R9 = log-sigma-3-0-mm-3D_glrlm_LowGrayLevelRunEmphasis.

Supplemental Table 4 The ROC analysis of three predictive models in training and test cohort.

| | | **Radiomic** | **Conventional** | **Combined** | $P_1$ | $P_2$ | $P_3$ |
|---|---|---|---|---|---|---|---|
| **Training** | **AUC** | 0.850 | 0.853 | 0.850 | 0.859 | 0.995 | 0.846 |
| | **95%*CI*** | 0.803~0.897 | 0.807~0.900 | 0.803~0.896 | | | |
| **Test** | **AUC** | 0.833 | 0.824 | 0.848 | 0.775 | 0.608 | 0.452 |
| | **95%*CI*** | 0.733~0.934 | 0.723~0.924 | 0.750~0.946 | | | |

AUC = area under the curve; *CI* = confidence index; $P_1$ = Radiomic vs Conventional; $P_2$ = Radiomic vs Combined; $P_3$ = Conventional vs Combined.

Supplemental Table 5 predictive results of models and radiologists in test cohort.

| | Radiomic | Conventional | Combined | A | B | A+R | B+R |
|---|---|---|---|---|---|---|---|
| **Accuracy** | 76.56% | 71.88% | 78.13% | 73.44% | 59.38% | 79.69% | 75.00% |
| **Sensitivity** | 82.35% | 82.35% | 91.18% | 61.76% | 50.00% | 79.41% | 82.35% |
| **Specificity** | 70.00% | 60.00% | 63.33% | 86.67% | 70.00% | 80.00% | 66.67% |
| **PPV** | 75.68% | 70.00% | 73.81% | 84.00% | 65.38% | 81.82% | 73.68% |
| **NPV** | 77.78% | 75.00% | 86.36% | 66.67% | 55.26% | 77.42% | 76.92% |
| **MR** | 30.00% | 40.00% | 36.67% | 13.33% | 30.00% | 20.00% | 33.33% |
| **MDR** | 17.65% | 17.65% | 8.82% | 38.24% | 50.00% | 20.59% | 17.65% |

A, the first binary diagnosis of the senior radiologist with 10-year experience in thoracic imaging diagnosis in test cohort; B, the first binary diagnosis of the junior radiologist with 2-year experience in medical imaging diagnosis in test cohort; A+R, the second binary diagnosis of radiologist A in test cohort with the aid of radiomic predictive model; B+R, the second binary diagnosis of radiologist B in test cohort with the aid of radiomic predictive model; PPV = positive predictive value; NPV = negative predictive value; MR = misdiagnosis rate; MDR = missed diagnosis rate.

Supplemental table 6 Distribution of each features in training and test cohort.

| | **Total** | **Training cohort** | **Test cohort** | ***P*** |
|---|---|---|---|---|
| | 322 | 258 | 64 | |
| **Pathology** | | | | |
| **AIS/MIA** | 150 (46.6%) | 120 (46.5%) | 30 (46.9%) | 0.958 |
| **IVA** | 172 (53.4%) | 138 (53.5%) | 34 (53.1%) | |
| **Gender** | | | | |
| **Male** | 82 (25.5%) | 70 (27.1%) | 12 (18.8%) | 0.168 |
| **Female** | 240 (74.5%) | 188 (72.9%) | 52 (81.3%) | |
| **Age(year)** | 53 (25, 78) | 53 (25, 78) | 54 (30, 72) | 0.618 |
| **Smoking history** | | | | |
| **Never** | 289 (89.8%) | 230 (89.1%) | 59 (92.2%) | 0.474 |
| **Current or ever** | 33 (10.2%) | 28 (10.9%) | 5 (7.8%) | |
| **Diameter (cm)** | 0.94 (0.24, 2.94) | 0.94 (0.24, 2.94) | 0.93 (0.34, 2.04) | 0.796 |
| **Density (HU)** | -571.0 (-829.2, -122.5) | -583.0 (-829.2, -122.5) | -525.8 (-763.2, -179.8) | 0.138 |
| **R1** | $7*10^{-3}$ ($1*10^{-3}$, $82*10^{-3}$) | $7*10^{-3}$ ($1*10^{-3}$, $82*10^{-3}$) | $6*10^{-3}$ ($2*10^{-3}$, $41*10^{-3}$) | 0.766 |
| **R2** | 8.1 (3.9, 10.0) | 8.1 (3.9, 10.0) | 8.3 (5.2, 10.0) | 0.823 |
| **R3** | 6.3 (3.0, 7.5) | 6.3 (3.0, 7.5) | 6.4 (4.2, 7.4) | 0.548 |
| **R4** | $5*10^{-3}$ ($1*10^{-3}$, $83*10^{-3}$) | $5*10^{-3}$ ($1*10^{-3}$, $83*10^{-3}$) | $4*10^{-3}$ ($1*10^{-3}$, $28*10^{-3}$) | 0.662 |
| **R5** | 8.4 (1.6, 104.8) | 8.2 (1.6, 104.8) | 9.0 (2.5, 45.6) | 0.753 |
| **R6** | 7.4 (1.6, 93.7) | 7.4 (1.6, 93.7) | 7.4 (1.9, 38.7) | 0.718 |
| **R7** | 4.3 (1.0, 19.4) | 4.2 (1.0, 19.4) | 5.0 (1.0, 17.) | 0.655 |
| **R8** | 110.6 (5.8, 1874.6) | 108.0 (5.8, 1874.6) | 121.9 (16.6, 786.9) | 0.945 |
| **R9** | $43*10^{-3}$ ($4*10^{-3}$, 687*10-3) | $43*10^{-3}$ ($4*10^{-3}$, 687*10-3) | $39*10^{-3}$ ($4*10^{-3}$, 488*10-3) | 0.452 |

We set training and test cohort randomly by a ratio of 4:1. For each feature, there was no significant difference between pGGN-like AIS/MIA and IVA. Continuous variables were represented in median (minimum, maximum) without any special statement. pGGNs = pure ground glass nodules; AIS =

adenocarcinoma in situ; MIA = minimally invasive adenocarcinoma; IVA = invasive adenocarcinoma; R1 = wavelet-LLL_glcm_MaximumProbability; R2 = original_glcm_JointEntropy; R3 = wavelet-LLL_glcm_SumEntropy; R4 = original_glcm_JointEnergy; R5 = log-sigma-1-0-mm-3D_glszm_GrayLevelNonUniformity; R6 = wavelet-LLH_glszm_GrayLevelNonUniformity; R7 = log-sigma-3-0-mm-3D_glszm_GrayLevelNonUniformity; R8 = original_glszm_SizeZoneNonUniformity; R9 = log-sigma-3-0-mm-3D_glrlm_LowGrayLevelRunEmphasis.
